# Image guided sacroiliac joint corticosteroid injections in children: an 18-year single-center retrospective study

**DOI:** 10.1186/s12969-020-00435-8

**Published:** 2020-06-17

**Authors:** Racha Chamlati, Bairbre Connolly, Ronald Laxer, Jennifer Stimec, Jyoti Panwar, Shirley Tse, Prakash Muthusami, Joao Amaral, Michael Temple, Dimitri A. Parra

**Affiliations:** 1 Division of Image Guided Therapy, Department of Diagnostic Imaging, The Hospital for Sick Children, University of Toronto, 555 University Avenue, Toronto, ON M5G 1X8 Canada; 2 Division of Rheumatology, Department of Paediatrics, The Hospital for Sick Children, University of Toronto, Toronto, Canada; 3 Department of Diagnostic Imaging, The Hospital for Sick Children, University of Toronto, Toronto, Canada

**Keywords:** Sacroiliac joints, Corticosteroid injections, Juvenile idiopathic arthritis, Children

## Abstract

**Background:**

Sacroiliitis is commonly seen in enthesitis-related arthritis (ERA), a subtype of juvenile idiopathic arthritis (JIA). Sacroiliitis is characterized by the inflammation of the sacroiliac (SI) joints (+/− adjacent tissues). The treatment options include systemic therapy with or without corticosteroid SI joint injections. Image guided SI joint injections are frequently requested in pediatric patients with sacroiliitis.

The purpose of this study was to evaluate the feasibility and efficacy of SI joint injections in children with sacroiliitis.

**Methods:**

A retrospective study of patients referred to Interventional Radiology (IR) for SI joint corticosteroid injections (2000–2018). Clinical information was collected from Electronic Patient Charts and procedural details from PACS. Efficacy was determined clinically, by MRI, or both when available.

**Results:**

50 patients (13.8 years; M:F = 35:15) underwent image-guided SI joint corticosteroid injections. Most common indications were JIA (84%) and inflammatory bowel disease (14%). 80% had bilateral injections. 80% were performed under general anesthesia and 20% under sedation. The corticosteroid of choice was triamcinolone hexacetonide in 98% of patients. Needle guidance and confirmation was performed using CT and fluoroscopy (54%), Cone Beam CT (CBCT, 46%), with initial ultrasound assistance in 34%. All procedures were technically successful without any complications. 32/50 patients had long-term follow-up (2 years); 21/32 (66%) had clinical improvement within 3-months. Of 15 patients who had both pre- and post-procedure MRIs, 93% showed short-term improvement. At 2 years, 6% of patients were in remission, 44% continued the same treatment and 47% escalated treatment.

**Conclusion:**

Image-guided SI joint injections are safe and technically feasible in children. Imaging modalities for guidance have evolved, with CBCT being the current first choice. Most patients showed short-term clinical and imaging improvement, requiring long-term maintenance or escalation of medical treatment.

## Background

Juvenile idiopathic arthritis (JIA) is the most common pediatric rheumatic disease and cause of arthritis in children, with an incidence of 1 to 22 in 100,000 and a prevalence of 7 to 150 in 100,000 [1]. JIA is defined as persistent arthritis for at least 6 weeks, presenting before 16 years of age, of unknown etiology after excluding other causes [2]. The International League of Association for Rheumatology (ILAR) classifies JIA into seven subtypes: systemic arthritis, oligoarthritis (persistent and extended), polyarthritis rheumatoid factor negative, polyarthritis rheumatoid factor positive, enthesitis-related arthritis (ERA), psoriatic arthritis, and undifferentiated arthritis [3, 4]. An important goal in JIA is early recognition and management to achieve inactive disease, maintain remission, and prevent irreversible joint damage and deformities [2].

Sacroiliitis can be seen in JIA, especially ERA, and is characterized by the inflammation of the sacroiliac joint and adjacent tissues. Sacroiliitis is a common feature of spondyloarthritis (SpA) with incidences that range from 20% in SpA related to inflammatory bowel disease, to 100% in ankylosing spondylitis [5]. Although frequently asymptomatic, sacroiliitis can present as lower back or buttock pain aggravated with movements, such as rising to stand, walking, running, and climbing. If untreated, it may lead to fusion of the SI joints. Sacroiliitis should be suspected if there is a history of inflammatory back pain or demonstration of positive SI provocation tests [6]. MRI is considered the gold standard for imaging of SI joints with a high sensitivity and specificity compared to plain radiography and bone scanning respectively [7, 8]. It demonstrates early features of inflammation and effusion, as well as chronic changes such as sclerosis, erosions, ankylosis and bone marrow changes. Although plain radiography can still be accepted if patients fulfill the New York criteria for sacroiliitis [7, 8], it can lead to a delay in diagnosis as x-rays are often normal at disease onset [9].

Multiple medications are used to treat patients with JIA, specifically those with ERA, including nonsteroidal Anti-Inflammatory Drugs (NSAIDs), Disease-Modifying Antirheumatic Drugs (DMARDs), corticosteroids and biologic agents including anti-tumor necrosis factor-alpha (anti-TNFα) inhibitors [10]. There are insufficient comparison studies on the efficacy of different treatment plans for the management of sacroiliitis in the pediatric population. NSAIDs are found to be effective in the short term, for direct symptomatic relief and are particularly effective for enthesitis in children [11]. Continuous use of NSAIDs in adults has been shown to achieve improvement radiologically, and to slow disease progression, however, their efficacy has not been determined in children [11]. An emerging trend is the use of anti-TNFα inhibitors, specifically etanercept, adalimumab and infliximab. They have shown exceptional clinical results and can be used as first line treatment for JIA patients with axial involvement [11]. Precautions with anti-TNFα inhibitors are required given the associated risk of infections, hypersensitivity reactions, psoriasis, demyelination and malignancy [11].

Another therapeutic option for arthritis in children is intra-articular (IA) corticosteroid injections, providing rapid improvement and with minimal systemic risks in comparison with systemic corticosteroids [12]. Potential risks of IA injections include infection, bleeding, skin atrophy, hypopigmentation, chemical irritation and calcium deposits and focal demineralization [12]. IA injections are often used in conjunction with systemic therapy at presentation or during the course of treatment. Corticosteroid injections (IA and tendon sheath) are performed by many paediatric interventional radiology (IR) practices using image guidance [2]. Image guided SI joint injections have been performed in our center for more than 15 years. In the context of new systemic therapies, the current role of IA injections in the treatment of pediatric patients with sacroiliitis is still to be determined. The aim of this retrospective study was to review the feasibility and efficacy of image guided corticosteroid SI joint injections in children.

## Methods

Research Ethics Board approval was obtained. This retrospective study was conducted at a pediatric tertiary care hospital. The study population included patients referred to IR for SI corticosteroid joint injections (Jan 2000 – Jan 2018). Patient demographics and clinical histories were collected from the Electronic Patient Charts (EPC). Procedural details and imaging were collected from Picture Archiving and Communication System (PACS, GE Milwaukee, USA).

## Technique

**Fig. 1:**
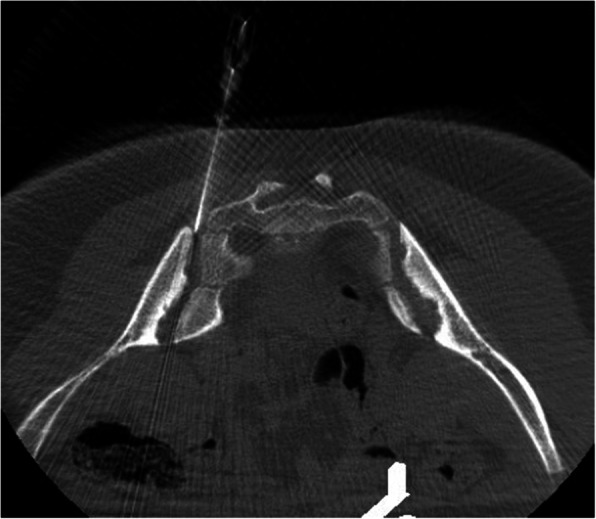
Cone beam CT guided SI joint injection w/o contract

Referral for SI joint injections were done by the rheumatology team. Sacroiliitis was diagnosis based on clinical symptoms, associated with consistent findings in MRI. Sedation or anesthetics were given based on patient preferences and cooperation, which was assessed by the nurse or anesthesiologist, as well as procedure details (e.g. number of concomitant joint injections). A staff pediatric interventionalist performed the procedures under image guidance. The interventional radiologist performing the injection obtained informed consent. Procedures were performed with the patient in the prone position and using a sterile technique. Needle guidance and positioning within the joint was performed and confirmed with CT fluoroscopy, or Cone Beam CT (available since 2010), depending on availability and/or radiologist preference (Fig. 1). Cone beam CT is a modality in which cross sectional images are generated from a flat panel C arm in the interventional radiology room. The images generated have a similar quality to conventional CT and allow procedure guidance with less radiation exposure. Ultrasound (US) was occasionally used, according to radiologist preference as well. The needle was placed in the lower third of the SI joint. The corticosteroid was prescribed by the rheumatology team based on the patient’s body weight. Triamcinolone hexacetonide was used in most cases and the doses are as follow: 10 to 20 Kg: 20 mg; more than 20 to 40 Kg: 30 mg; more than 40 Kg: 40 mg. In the case of triamcinolone acetonide the doses are 40, 60 and 80 mg respectively. The corticosteroid injection was followed by a similar volume of lidocaine 1%. Most of the time, no contrast was used due to the small joint space. A sterile dressing was applied to the skin. The referring rheumatologist was informed of the outcome of the procedure. Post-procedure, the patients were transferred to the post anesthesia care unit (PACU) and discharged home 2 hours later.

The efficacy of the SI corticosteroid injection was assessed both clinically and by MR imaging. Patients with incomplete data and those with IBD were excluded for this assessment. Medical records were reviewed for peri- and post-procedural complications. Clinical outcomes were based on the information available in clinical notes and assessed at two time periods, within 3 months (*short term*) and 2 years post injection (*long term*). Short term response was classified as *Good* or *Poor;* patients rendered asymptomatic or experiencing a decrease in severity of symptoms such as pain at the injected joints within a 3 month period as per documented in the chart were classified as having a good response to treatment; patients experiencing the same or an increase in severity of symptoms within a 3 month period were classified as having a poor response to treatment. Two radiologists (JS, JP) assessed imaging response in patients with both pre- and post-injection MRI by using the Spondyloarthritis Research Consortium of Canada (SPARCC) scoring system. Both observers were blinded to clinical information and timing of the studies and they worked independently. All examinations were performed on a 1.5-T MRI system (Magnetom Avanto, Siemens, Erlangen, Germany), using a dedicated surface coil system and included oblique coronal short tau inversion recovery (STIR) imaging (repetition time msecTR/TE/TIecho time msec of 2250/69/x msec, with a field of view of 25 cm, slice thickness of 4 mm with a slice gap of 4.5 mm) of the SI joints. Scoring was performed on six consecutive oblique coronal slices, covering most of the cartilaginous and synovial portion of the joint, by utilizing three MR indices: 1) presence of bone marrow edema, 2) extent or depth of edema and 3) intensity of edema [13, 14]. Patients experiencing a decrease in SPARCC score post-injection were classified as having a good response to treatment, whereas those with an increase in score were classified as having a poor response to treatment.

Clinical status 2 years post-injection, long term response was categorized as inactive disease (remission and off medications) and ongoing disease activity that was then divided into 3 subcategories: decreased (decrease dose/frequency or removal of drug), maintained (unchanged dose/frequency of drug) or escalated (increase dose/frequency or addition of drug).

## Results

### Patient population

**Fig. 2:**
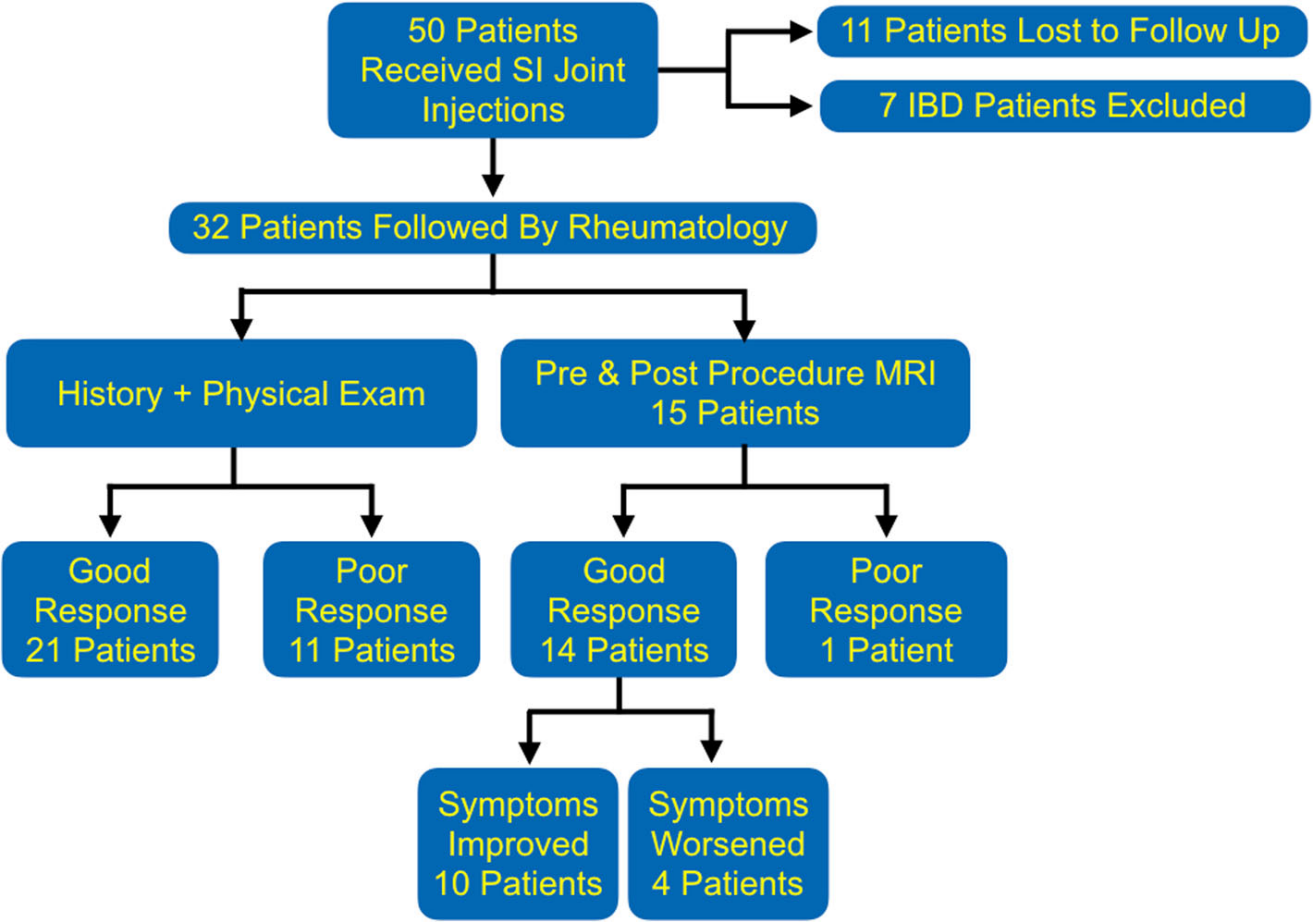
Patient flow chart

Fifty patients with sacroiliitis underwent SI joint injections during the study period and all injection were technically successful. Thirty-five (70%) were male and 15 were female (30%), ranging from 8 to 18 years old with a median age of 14 years, IQR of 4. 32/50 (64%) had a two year follow up and were included in the clinical response assessment; 15/50 (30%) had both pre- and post-injection MRI imaging and were included in the imaging assessment; 15/50 (30%) had both clinical and imaging assessment performed (Fig. 2).

The most common etiology was JIA (84%) followed by inflammatory bowel disease (14%). 26 patients (52%) were HLA B27 positive, 17 patients (34%) were HLA B27 negative and 7 patients (14%) were not tested. Twenty-two out of the 26 HLA B27+ patients (85%) were diagnosed with ERA, 1 (4%) had psoriatic arthritis and 3 (11%) had IBD (Table 1). 3/50 (6%) required two SI joint injections during the study period. Injections were performed bilaterally in 80% and unilaterally in 20% (40% left and 60% right). General anesthesia was used in 80% and sedation in 20% (14% nurse sedation, 6% anesthesiologist). Triamcinolone hexacetonide was used in 49 patients (98%) as this is our preferred intra-articular corticosteroid. Triamcinolone acetonide was employed in 1 patient (2%) due to shortage of triamcinolone hexacetonide in the country. A 22 G Chiba needle was most commonly used (17 patients, 40%).The other needles used were: 22 Spinal (16.6%), 25 Spinal (38.1%), 20 Chiba (2.4%) and 18 Trocar (2.4%). The greater part of the patients (18 patients, 36%) received a dose of 20 mg (1 ml). CT fluoroscopy (2000–2016) was used for image guidance and confirmation in 27 patients (54%), with adjunctive US in 12/27; Cone Beam CT (2010–2018) was employed in 23 patients (46%), with adjunctive US in 5/23. All procedures were technically successful without any complications.

**Table 1:** Patient demographics

Mean Age (years)	13.8 (8–18)
Gender	F: 15 (30%); M: 35 (70%)
**HLA B 27 Status**	**Number of Patients (%)**
Positive	26 (52)
Negative	17 (34)
Not Done	7 (14)
**Patient Diagnosis**	**Number of Patients (%)**
Juvenile Idiopathic Arthritis	42 (84)
Inflammatory Bowel Disease	7 (14)
Isolated Sacroiliitis	1 (2)

**Fig. 3:**
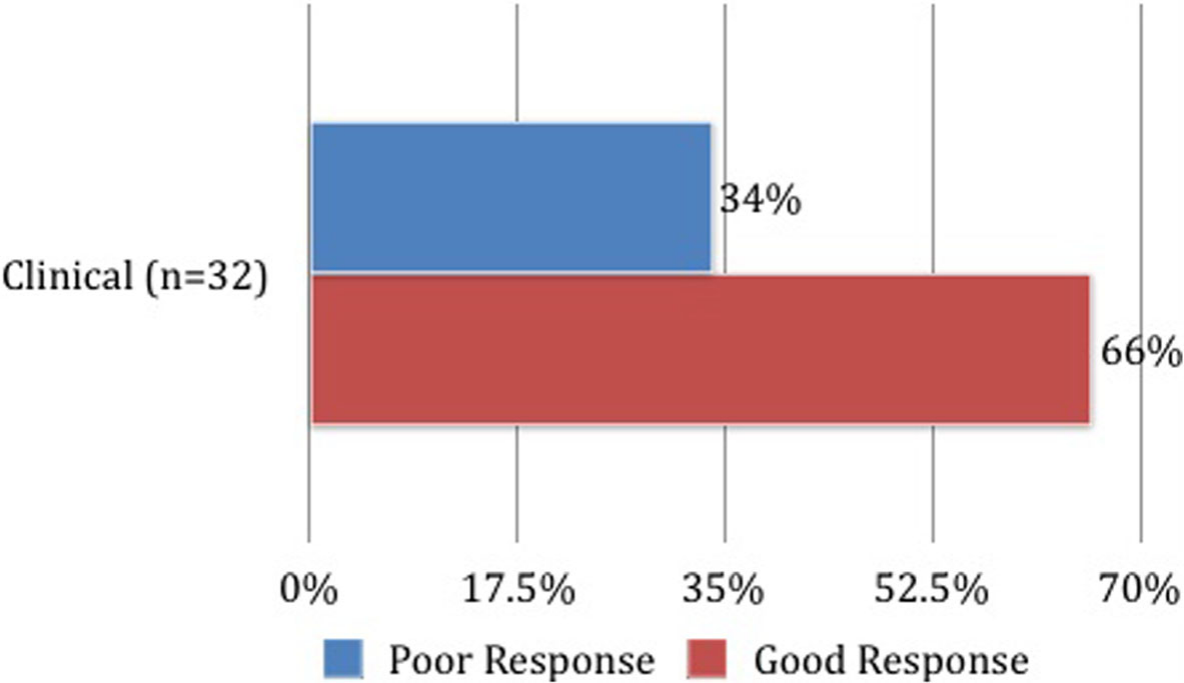
Post injection short term assessment

Of the 32 patients with a short (3-month) and long-term (2 year) follow-up, 18 patients (56%) had only SI joint involvement, while the remaining 14 patients (44%) had multiple joints involved. In the 3-month follow up period, 21 (66%) patients improved clinically and had no residual SI joint tenderness, whereas 11/32 (34%) remained symptomatic with active sacroiliitis (Fig. 3). Within two years post injection, remission was achieved in 2/32 patients (6%); treatment was reduced in 1/32 patient (3%), maintained in 14 patients (44%) and escalated in 15 patients (47%), with 13/15 starting on biologics. Among the 13 patients who were escalated to biologics, 7 (54%) had isolated sacroiliitis and 6 (46%) had multiple joint involvement.

**Fig. 4:**
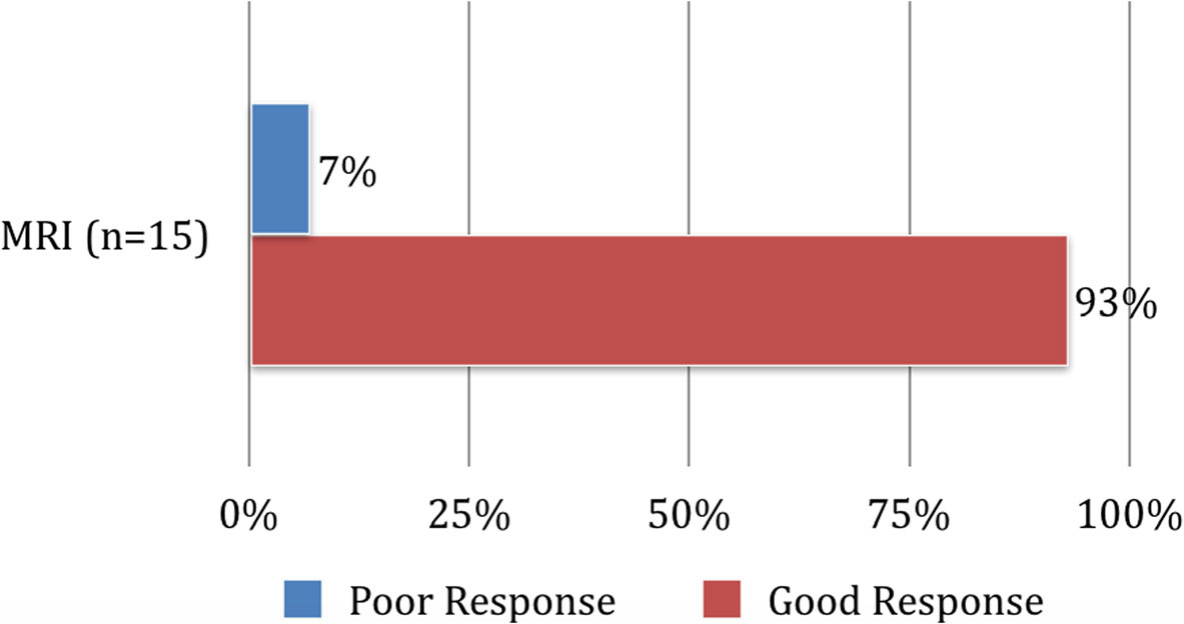
Post injection SPARCC score assessment

Fifteen out of the 32 patients had a pre- and post-procedure MRI with a mean of 9.4 months (range 0.25 to 33 months) post injection. 11/15 (73%) had isolated sacroiliitis and 4/15 (27%) had multiple joint involvement. On post-procedure MRI, 14/15 (93%) showed a decrease in their SPARCC score, 2 of whom (14%) were on biologics prior to the injection, 10/14 (66.7%) improved clinically and the remaining 4 patients (26.7%) reported worsening of symptoms (Fig. 4); 1/15 (6.6%) had a higher SPARCC score. The median change in SPARCC score was found to be − 14.5 with an interquartile range of 12.5. Out of the 14 patients with a decrease in SPARCC score, 12 (86%) met the minimal clinically important difference of 2.5.

As mentioned before, In the 2-year follow up period, 13 patients had an escalation of treatment starting biologic agents, with a mean initiation time of 6.11 months (range 0.5 to 18 months) post-injection. When taking into account joint involvement, in the 11 patients with isolated sacroiliitis, 1 (9%) achieved remission, 1 (9%) reduced treatment, 4 (36%) remained on the same treatment plan and 5 (46%) escalated. Among the 4 patients with multiple joint involvement, 3 (75%) remained on the same treatment plan and 1 (25%) escalated to biologics. Table 2 ilustrates the long term follow up in the group of patients in whom a change of SPARCC score was assessed.

**Table 2:** Long term management Vs. MRI findings

	Decrease SPARCC	Increase SPARCC
**Decrease Treatment**	1 (6.7%)	0
**Same Treatment**	6 (40%)	1 (6.7%)
**Escalated Treatment**	6 (40%); 5 (33%) started biologics	0
**Remission**	1 (6.7%)	0

## Discussion

Sacroiliitis is a prominent clinical manifestation of the ERA sub-type of JIA and is frequently associated with HLA B27 positivity. It can be treated medically or by SI corticosteroid joint injections. The management of patients with JIA requires a multidisciplinary approach as shown in this study in which rheumatology, diagnostic imaging and interventional radiology worked together to provide diagnosis, treatment and follow up. Prior to image guided techniques, corticosteroid injections were performed by palpation of anatomical landmarks. However, this technique lacked specificity or efficiency due to the complexity of the sacroiliac joint anatomy [15]. With evolving imaging technology, image guided techniques have become increasingly available. Historically, fluoroscopy was employed. With the advent of cross sectional imaging, conventional CT or cone beam CT has been used to guide needle placement and ensure successful intra-articular injections [15]. US has also been utilized for needle guidance, on its own or combined with cross sectional imaging [16]. More recently there are reports of MRI guided injections [17]. The use of three needles has been reported in the literature, in the inferior, middle and superior aspects of the joint [17], however currently only one needle in the inferior or middle synovial portion of the joint is preferred [18]. Although two different cross sectional imaging modalities were used in this study, all cases were successfully completed without any peri- or post-procedure complication. US assisted in approximately one third of the SI joint injections. From 2000 to 2009, CT fluoroscopy were the only cross sectional imaging option available in this IR practice. In 2010, Cone Beam CT became available and provided satisfactory image guidance for the procedure. It eventually replaced CT fluoroscopy in 2016 due to its adequate performance, ease of use and recognized radiation dose reduction, as has been demonstrated in the literature [19, 20]. The corticosteroid currently used is triamcinolone hexacetonide as studies have shown that it is more effective than hydrocortisone succinate and triamcinolone acetonide in reducing pain and joint inflammation [21, 22].

Similar to the benefits of injections in other joints, corticosteroids relieved the symptoms (especially pain) with SI joint inflammation in the majority of the patients. This was assessed in two ways: clinically by patient history/physical exam, and on imaging by assessing and scoring MRIs before and after the procedure. Most of the patients with a decrease of inflammation on post injection MRI also reported symptomatic improvement and met the MCID in the SPARCC SIS of 2.5 [23]. An interesting finding is that a group of patients despite the imaging improvement had worsening of symptoms. The reason why this happens is currently unclear to us and warrants further studies.

Clinical improvements were not sustained in the majority of patients who received joint injections. Within 2 years, only two patients achieved remission and one patient a reduction in treatment. The remainder stayed on the same management plan or escalated to a higher level of medical therapy (eg. biologic agents) within a year. More than half of the patients who transitioned to biological agents had exclusively SI joint involvement.

There are several limitations with this retrospective study. Disease severity may have been underestimated as patient history and complaints were limited to those recorded in the rheumatology clinic notes. Radiological reports may not have captured all procedural details or difficulties encounter during SI joint injections, resulting in an underestimate. Some patients were transferred to adult facilities once they came of age or were simply lost to follow-up. Follow-up MRIs were not consistently performed on a protocol basis, but variably depending on clinical indication. Due to the complexity of the management of sacroiliitis in JIA patients, a comparison of monotherapy with corticosteroid injections to medical management alone was not possible.

## Conclusion

Image guided SI joints injections are safe and feasible in pediatric patients. With the evolution of fluoroscopic systems, CBCT has replaced conventional CT as the imaging modality of choice. In our cohort of patients, intra-articular injections provided short-term symptom relief, but in the longer term, most of the patients were maintained or escalated to systemic medical therapy within months to a year. The lack of standardized management and follow up protocols makes it difficult to analyze the clinical evolution of these patients. Collaboration between rheumatology, diagnostic and interventional radiology is beneficial in their management. The role of corticosteroid SI joint injections in the context of new systemic therapies requires further investigation.

## Abbreviations

ERA: enthesitis-related arthritis
JIA: juvenile idiopathic arthritis;
SI: sacroiliac
IR: interventional radiology
CBCT: cone beam CT
ILAR: International League of Association for Rheumatology
SpA: spondyloarthritis
NSAIDs: nonsteroidal Anti-Inflammatory Drugs
DMARDs: Disease-Modifying Antirheumatic Drugs
anti-TNFα: anti-tumor necrosis factor-alpha
IA: intra-articular
EPC: Electronic Patient Charts PACS: Picture Archiving and Communication System; US: Ultrasound; PACU: post anesthesia care unit; IBD: inflammatory bowel disease; SPARCC: Spondyloarthritis Research Consortium of Canada; STIR: short tau inversion recovery; MCID: minimal clinically important differences; SPARCC SIS: Spondyloarthritis Research Consortium of Canada sacroiliac joint inflammation score.
